# Supplementary material for: Skeletal muscle cells opto-stimulation by intramembrane molecular transducers
Source: Commun Biol. 2023 Nov 11;6:1148. doi: 10.1038/s42003-023-05538-y (PMC10640616; doi:10.1038/s42003-023-05538-y)
Supplement: Supplementary file 2 — Supplementary Information [file 42003_2023_5538_MOESM2_ESM.pdf]

Supporting Information

Skeletal muscle cells opto-stimulation by intramembrane molecular transducers

Skeletal muscle cells opto-stimulation by intramembrane molecular transducers

Ilaria Venturino[1,2], Vito Vurro [2], Silvio Bonfadini [2], Matteo Moschetta [2], Sara Perotto [2], Valentina Sesti [2,3], Luigino Criante [2], Chiara Bertarelli [2,3], Guglielmo Lanzani [*,1,2]

1. Dipartimento di Fisica, Politecnico di Milano, Milano, Italy

2. Center for Nano Science and Technology, Istituto Italiano di Tecnologia, Milano, Italy

3. Dipartimento di Chimica Materiali e Ingegneria Chimica "Giulio Natta" Politecnico di Milano, Milano, Italy

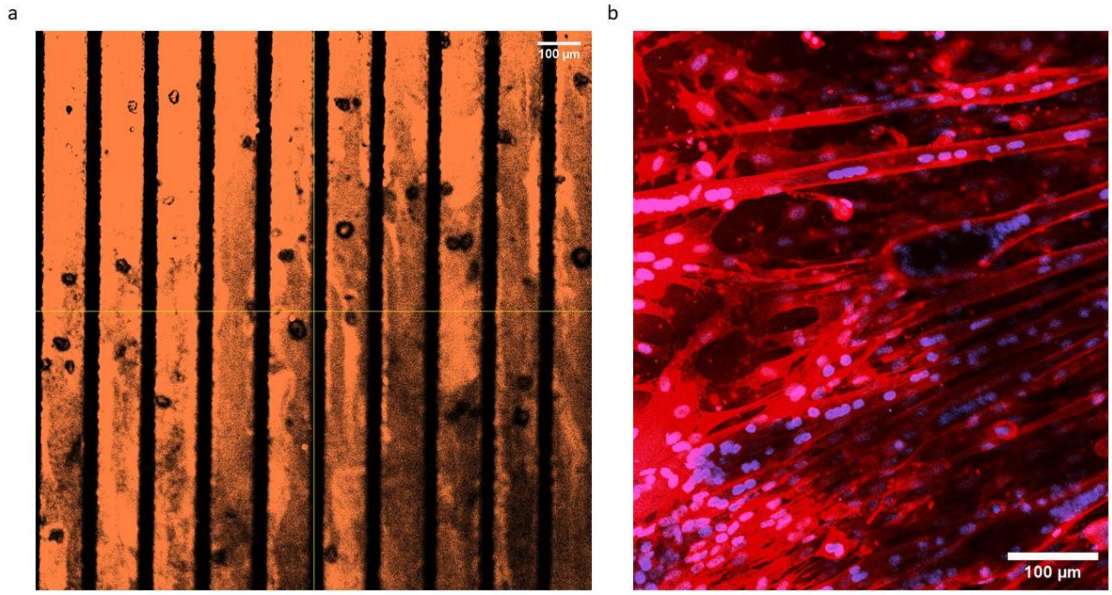

S1. Stamps and cells' growth. *a.) The pattern imprinted by the PDMS ablated stamps. The stamps have been covered with rhodamine B.*

b.) An example of the images used to evaluate the fusion index and. The nuclei are stained with Hoechest (blue) and the cells' membrane is stained with Deep Red CellMask (red). (r=3, n=5, f=10.)

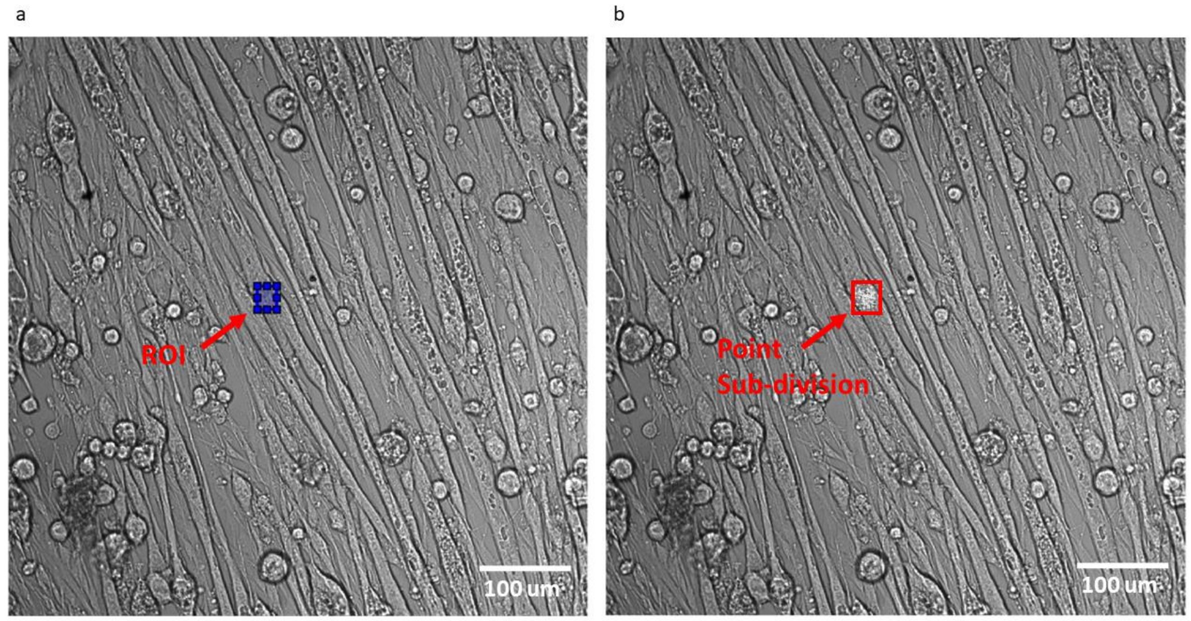

S2. Example of image produced by the Matlab vison recognition code. *a.) The region of interest selected by the user. b.) The withe points represent the sub-area of the ROI. The code evaluate the position of each single points and it use it to follow the movement of an object.*

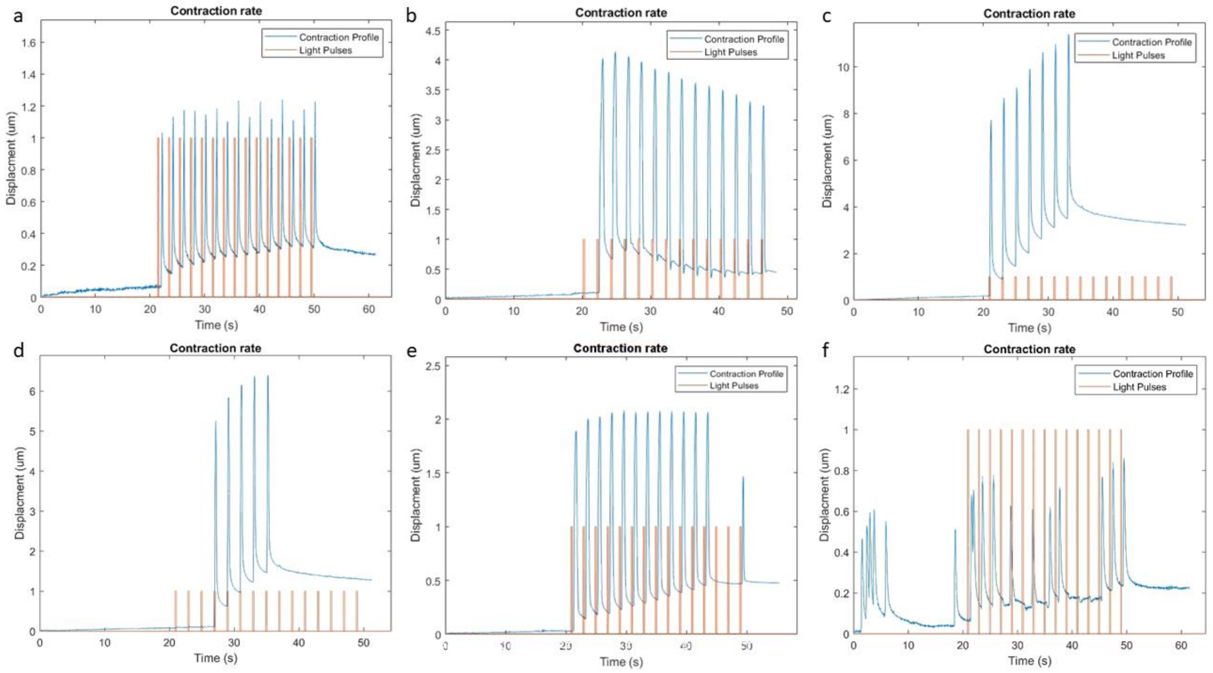

S3. Examples of different contraction path*. a.) Contraction profile that follow perfectly the light (casa A). b.) The contraction path starts after several seconds (case B). c.) The contraction path stops before the stimulation protocol (case C). d.) The myotubes start to contract after the beginning of the protocol and stop before its ends (case D). e.) During the stimulation, the myotubes stop for several second and then start again (case E). f.) The myotubes behave too randomly to be categorised in one of the previous classes (case F).*

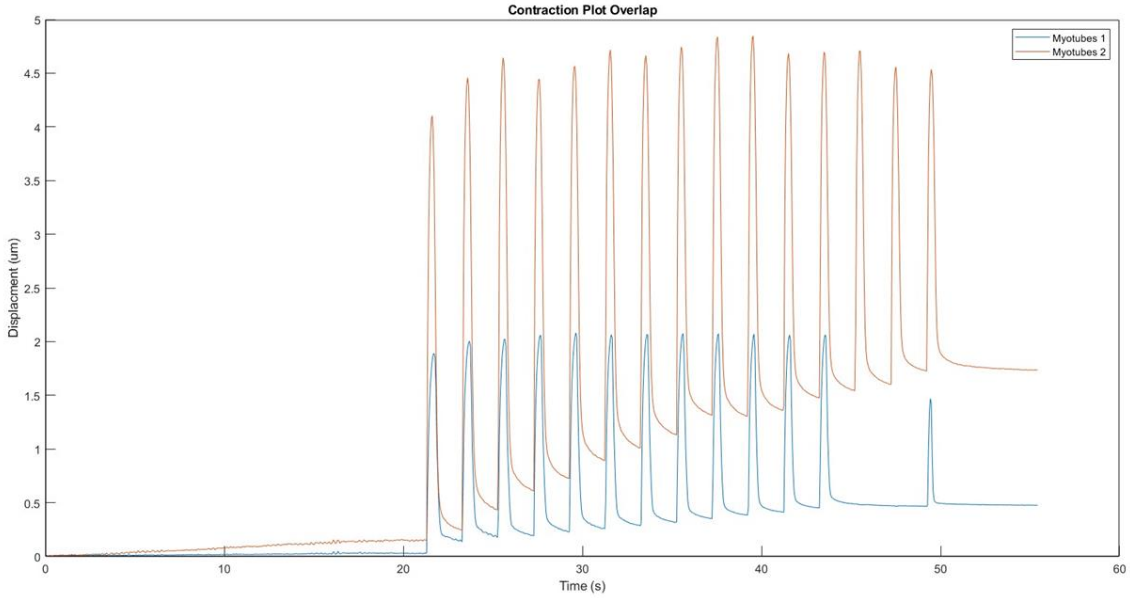

S4. Examples of two contracting myotubes from different cases. *Myotubes 1 (case E) and myotubes 2 (case A.) It is visible the deterministic effect of the light to the contraction profile of the analysed cells.*

Energy comparison

Our objective was to compare the energy absorbed by the system during the optical stimulation and the electrical stimulation. We know the delivered power density measured at the sample (P_{sample}) and the cross section of the molecule (σ=$2 * 10^{-15} cm^2$). We have also estimated the number of molecules internalized by the cells, $n = 10^{10} molecole * cm^{-2}$.

Eq.1 $\frac{\Delta E}{\Delta t} = \sigma n P_{sample}$

Through equation 1 we evaluate the energy absorb by time unit and area, and knowing that the duration of a pulse was Δt = 200 ± 0.1 ms, we could evaluate the energy absorbed by the highlighted area.

On the other hand, to evaluate the energy absorbed during the electrical stimulation we made some geometric assumptions. First of all, we assume that the field was almost uniform in the region between the two electrodes and in the region promptly below them, were the cells are located. To evaluate the resistance of the thin sheet of cells we used the resistivity of ρ=$5.78 \pm 1.4\ 10^{-1} \Omega m$, the distance between the two electrodes $d = 7 \pm 2 * 10^{-3}\ m$, the height of the myotubes $h = 1 * 10^{-5} m$ and the length of the electrode $l = 2.4 \pm 0.1 * 10^{-2} m$. The resulting resistance of the myotubes is equal to:

Eq. 2 $R = \frac{\rho d}{hl} = 1.2 \pm 0.6 * 10^4 \mathbf{\Omega}$

The power P can be obtained through the basic following equation:

Eq.3 $P = \frac{V^2}{R}$

To evaluate the energy we multiply the power by the duration of the electrical stimulus, Δt = 20 ± 0.1 ms. Then we normalize the energy by the area between the two electrodes given by $A = d * l$, that has a value of 1.69 $\pm$ 0.5 cm^2.

We could then compare the two energy absorb by the investigated region as the power density of the light increases or the voltage increases. In figure S5 we report the two trend line.

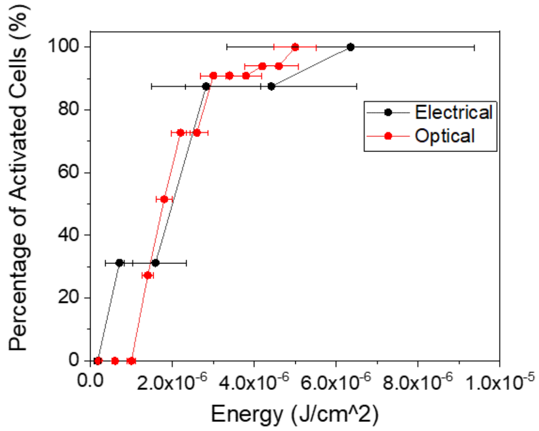

*S5. **Energy comparison.** The graph report the energy absorb by the cell when they are stimulate with light (red line) or with the electric field (black line).*

It is visible from the graph that the energy released to obtain the 100% of cells activation is little bit smaller when we look at the data of the optical stimulation, $5.0 \pm 0.5 * 10^{-6} J * cm^{-2}$ $compered\ to$ $6.4 \pm 3.0 * 10^{-6} J * cm^{-2}$.

Video List

Supplementary Video 1. The video reports the stimulation of myotubes at 0.5 Hz

Supplementary Video 2. The video reports the stimulation of myotubes at 1 Hz

Supplementary Video 3. The video reports the stimulation of myotubes at 2 Hz

Supplementary Video 4. The video reports the stimulation of a free standing device at 1 Hz
