# Supplementary material for: Skeletal muscle cells opto-stimulation by intramembrane molecular transducers
Source: Commun Biol. 2023 Nov 11;6:1148. doi: 10.1038/s42003-023-05538-y (PMC10640616; doi:10.1038/s42003-023-05538-y)
Supplement: Supplementary file 3 — Description of Additional Supplementary Files [file 42003_2023_5538_MOESM3_ESM.pdf]

Description of Additional Supplementary Files

File name: Supplementary Data
Description: All the data used in the figures.

File name: Supplementary Video 1
Description: The video reports the stimulation of myotubes at 0.5 Hz

File name: Supplementary Video 2
Description: The video reports the stimulation of myotubes at 1 Hz

File name: Supplementary Video 3
Description: The video reports the stimulation of myotubes at 2 H

File name: Supplementary Video 4
Description: The video reports the stimulation of a free standing device at 1 Hz
